# Local scale processes drive long‐term change in biodiversity of sandy beach ecosystems

**DOI:** 10.1002/ece3.3064

**Published:** 2017-05-25

**Authors:** Nicholas K. Schooler, Jenifer E. Dugan, David M. Hubbard, Dale Straughan

**Affiliations:** ^1^ Marine Science InstituteUniversity of California, Santa BarbaraSanta BarbaraCAUSA

**Keywords:** anthropogenic impacts, coastal, habitat loss, intertidal, macroinvertebrates, macrophyte wrack, marine, recovery, species richness, species–area curves

## Abstract

Evaluating impacts to biodiversity requires ecologically informed comparisons over sufficient time spans. The vulnerability of coastal ecosystems to anthropogenic and climate change‐related impacts makes them potentially valuable indicators of biodiversity change. To evaluate multidecadal change in biodiversity, we compared results from intertidal surveys of 13 sandy beaches conducted in the 1970s and 2009–11 along 500 km of coast (California, USA). Using a novel extrapolation approach to adjust species richness for sampling effort allowed us to address data gaps and has promise for application to other data‐limited biodiversity comparisons. Long‐term changes in species richness varied in direction and magnitude among beaches and with human impacts but showed no regional patterns. Observed long‐term changes in richness differed markedly among functional groups of intertidal invertebrates. At the majority (77%) of beaches, changes in richness were most evident for wrack‐associated invertebrates suggesting they have disproportionate vulnerability to impacts. Reduced diversity of this group was consistent with long‐term habitat loss from erosion and sea level rise at one beach. Wrack‐associated species richness declined over time at impacted beaches (beach fill and grooming), despite observed increases in overall intertidal richness. In contrast richness of these taxa increased at more than half (53%) of the beaches including two beaches recovering from decades of off‐road vehicle impacts. Over more than three decades, our results suggest that local scale processes exerted a stronger influence on intertidal biodiversity on beaches than regional processes and highlight the role of human impacts for local spatial scales. Our results illustrate how comparisons of overall biodiversity may mask ecologically important changes and stress the value of evaluating biodiversity change in the context of functional groups. The long‐term loss of wrack‐associated species, a key component of sandy beach ecosystems, documented here represents a significant threat to the biodiversity and function of coastal ecosystems.

## 1 INTRODUCTION

On a global scale, biodiversity is unequivocally considered to be declining due to species extinctions driven by climate change, development, and other human impacts (Butchart et al., 2010; Gonzalez et al., 2016; Hoegh‐Guldberg & Bruno, 2010; Pimm, Russell, Gittleman, & Brooks, 1995; Sala et al., 2000). However, biodiversity declines have not been consistently observed at local and regional spatial scales (Dornelas et al., 2014; Hautekèete et al., 2015; Sax & Gaines, 2003; Thomas, 2013; Vellend et al., 2013). As Earth's climate changes, determining whether and how biodiversity is decreasing and the processes responsible is the most pressing issue facing modern ecologists (Gonzalez et al., 2016).

Shifts in geographic ranges of individual species in response to climate change have already been described extensively (e.g., Burrows et al., 2011; Chen, Hill, Ohlemüller, Roy, & Thomas, 2011; Parmesan & Yohe, 2003; Schoeman, Schlacher, & Defeo, 2014). At the same time, human impacts can increase diversity at multiple scales through mechanisms including changing disturbance regimes (Devictor & Robert, 2009) and addition of exotic species (Bruno, Kennedy, Rand, & Grant, 2004). Despite a growing number of local scale long‐term biodiversity studies (see Dornelas et al., 2014; Vellend et al., 2013), major gaps in the understanding of biodiversity change outside of developed nations and for underrepresented biomes seriously impede our ability to accurately quantify biodiversity change across the planet (Gonzalez et al., 2016). For example, only a few studies have assessed long‐term change in biodiversity for coastal ecosystems on a regional scale (e.g., Elahi et al., 2015; Novoa, Talley, Talley, Crooks, & Reyns, 2016; Smith, Fong, & Ambrose, 2006; Zabin et al., 2013) and none of these studies have addressed diversity change for sandy beach ecosystems, which dominate shorelines globally making up ~70% of open coasts (Schoeman et al., 2014).

Understanding the responses of communities and ecosystems to climate forcing is critical for conservation (Gonzalez et al., 2016; Harley et al., 2006). Coastal ecosystems are expected to be particularly sensitive to sea level rise and warming as intertidal communities are exposed to extremes in abiotic conditions (Harley et al., 2006). Sandy beach ecosystems support diverse, endemic intertidal communities on a narrow strip of habitat between the land and sea (Dugan et al., 2010; McLachlan et al., 1995). Local scale anthropogenic drivers, including beach filling, grooming, armoring, off‐road vehicle (ORV) use, fishing, and recreation, have been shown to impact beach ecosystems (Defeo et al., 2009; Schlacher et al., 2007), but data on scales sufficient to detect regional or global scale biodiversity change are lacking for these underrepresented biomes (Dugan et al., 2010; Gonzalez et al., 2016; Schoeman et al., 2014).

Ecosystems and species are not expected to be equally vulnerable to climate change and other stressors (Pacifici et al., 2015). Ecological theory and experiments have shown that declines in biodiversity could depress ecosystem stability and function and increase invasibility, which could lead to higher species richness (Cardinale et al., 2012; Gamfeldt et al., 2015; Lefcheck et al., 2015; Tilman, Isbell, & Cowles, 2014). Analyses of overall biodiversity could mask important changes that may only be apparent in a subset of the community that shares specific ecological traits. Identifying the functional groups or taxa with traits that make them vulnerable to extinction could increase our ability to accurately detect meaningful change in community structure, identify the mechanisms responsible, and predict which aspects of biodiversity are most likely to be impacted by changes in specific environmental factors or processes (Elahi et al., 2015).

**Figure 1:**
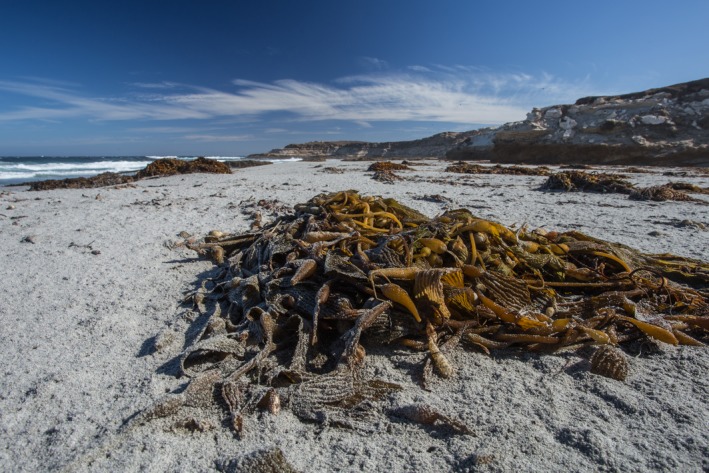
Macroalgal wrack, which is beach cast marine macrophytes, provides food and shelter for a large component of sandy beach macroinvertebrates. The large piles of wrack pictured here are composed primarily of giant kelp (*Macrocystis pyrifera*). The dense aggregation of burrows surrounding the wrack belong to low dispersal wrack‐associated species, talitrid amphipods (*Megalorchestia* spp.), which are the primary consumers of wrack on sandy beaches

Sandy beaches are characterized by low *in situ* primary production and subsidies of marine macrophytes cast ashore as wrack provide food and shelter to an important component of the overall intertidal community, wrack‐associated species (Figure 1). The diverse invertebrates associated with wrack (Figure 1) are highly sensitive to local impacts as well as climate change (Dugan, Hubbard, McCrary, & Pierson, 2003; Dugan, Hubbard, Rodil, Revell, & Schroeter, 2008; Hubbard, Dugan, Schooler, & Viola, 2014). Climate change is likely to affect the availability of wrack through multiple processes: warming is expected to decrease productivity of kelps in upwelling areas (Schiel & Foster, 2015), while losses of beach habitat due to sea level rise and erosion will reduce retention of wrack on beaches (Revell, Dugan, & Hubbard, 2011; Vitousek, Barnard, Limber, Erikson, & Cole, 2017). Wrack‐associated invertebrates, moreover, are characterized by limited dispersal ability and direct development (Grantham, Eckert, & Shanks, 2003) both of which likely reduce their resilience to disturbance (Dugan et al., 2003; Hubbard et al., 2014).

Here we evaluate the direction and magnitude of change in the biodiversity of sandy beach ecosystems in southern and central California, USA across more than three decades. We hypothesized that declines in overall species richness on beaches due to anthropogenic and climate change related impacts on habitat and food supply would be evident at both local and regional scales over this time span. We predicted that a vulnerable functional group, wrack‐associated invertebrates, would exhibit greater declines in species richness and be most sensitive to local and regional stressors due to their low dispersal ability and life histories and their dependence on a variable cross‐ecosystem subsidy.

## 2 MATERIALS AND METHODS

### 2.1 Biodiversity surveys

**Figure 2:**
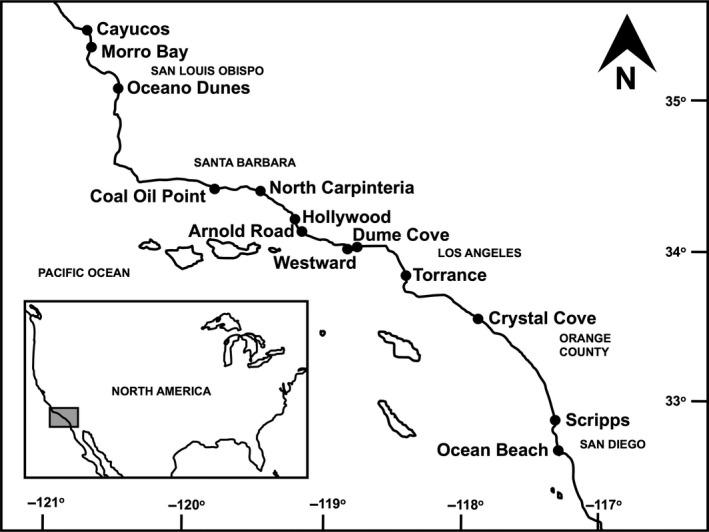
Map of the study region with names and locations of the sandy beach study sites (indicated by dots) surveyed on the coast of central and southern California, USA in the 1970s and in 2009–11 survey periods

Our 13 study beaches spanned ~500 km of coastline in California, USA, from Cayucos (35°26.058′N) to San Diego (32°44.523′N) (Figure 2, Table 1). All beaches could be classified as intermediate morphodynamic type for both study periods. Extensive intertidal macroinvertebrate surveys were conducted in the 1970s by Patterson (1974) and Straughan (1982). We used data from a total of 214 surveys conducted at the 13 beaches from 1969 to 1980 (hereafter 1970s surveys) (Appendix S1). To address the lack of complete datasets on species abundance and biomass at all 13 sites, we relied on cumulative species lists (Table S1) and area sampled data compiled for each site for our comparisons between survey periods. We compiled cumulative lists of overall, wrack‐associated, and low dispersal wrack‐associated species for each site from Patterson (1974) and Straughan (1982). We calculated a cumulative sampling area for each study beach using the number of surveys conducted, active intertidal zone widths (distance from the 24‐hr high tide line to the low swash limit), and sampling design for each survey based on Straughan (1982). We estimated the sampling area for wrack‐associated invertebrates by calculating the area of the upper‐beach zone where wrack‐associated species are found for each survey. This zone was smaller than the active intertidal zone and covered from the 24‐hr high tide line down to the lowest sample containing wrack‐associated species (Dugan, Hubbard, & Quigley, 2013).

**Table 1:** The names, coordinates of the basepoints (from Straughan, 1982), years surveyed, cumulative active intertidal sampling area, and number of surveys conducted for each survey period for the 13 study beaches arranged from north to south. The numbers in parentheses represent the number of proportional area surveys. A total of 249 surveys were conducted in the 1970s (214 surveys) and 2009–11 (35 surveys). Collectively the repeated surveys from the 1970s sampled more than three times as much active intertidal area (226 m^2^) as the 2009–11 surveys (71 m^2^). Direct beach alteration activities (grooming, beach fill, and ORV use) are listed by study beach. The number of surveys using the proportional area design during the 2009–11 surveys appears in parentheses. Dredge pipes were stretched across the upper‐beach habitat at one beach indicated by an asterisk in the Beach fill column

Beach name	Coordinates	Years surveyed	1970s		2009–11		Direct beach alteration		
			Area (m^2^)	Surveys	Area (m^2^)	Surveys	Grooming	Beach fill	ORV use
Cayucos	35°26′06.2″N, 120°53′18.7″W	1973–74; 2009–10	10.1	7	8.6	3 (2)	No	No	No
Morro Bay	35°22′33.8″N, 120°51′50.8″W	1973–74; 2009	12.5	8	6.5	2 (1)	No	Both*	No
Oceano Dunes	35°02′14.6″N, 120°37′58.5″W	1974; 2010	4.2	3	2.0	1 (1)	No	No	1970s
Coal Oil Point	34°24′33.2″N, 119°52′09.9″W	1969–78; 2009–11	34.9	52	7.0	6 (5)	No	No	No
North Carpinteria	34°23′29.4″N, 119°31′18.1″W	1970–71, 73–75, 78; 2009	36.6	35	4.5	2 (1)	No	No	No
Hollywood	34°10′13.1″N, 119°13′59.7″W	1971–74, 78; 2009	34.7	28	1.3	1 (1)	Both	Both	No
Arnold Road	34°07′17.5″N, 119°09′37.6″W	1971–74, 78; 2009–11	38.6	24	12.5	6 (4)	No	No	1970s
Westward	34°00′10.9″N, 118°48′34.4″W	1975–78; 2009–10	5.8	8	5.0	3 (2)	Both	Both	No
Dume Cove	34°00′21.8″N, 118°48′06.5″W	1971; 2011	2.0	6	2.1	1 (0)	No	No	No
Torrance	33°49′09.8″N, 118°23′25.1″W	1974–80; 2009	11.7	16	0.9	1 (1)	Both	Both	No
Crystal Cove	33°34′41.2″N, 117°50′52.3″W	1970–71; 2011	5.1	7	3.1	1 (0)	No	No	No
Scripps	32°51′49.2″N, 117°15′16.7″W	1975–78; 2009–11	17.9	10	14.5	6 (5)	No	No	No
Ocean Beach	32°51′52.3″N, 117°15′10.3″W	1975–78; 2009–10	12.1	10	2.9	2 (2)	Both	Both	No

We resurveyed intertidal macroinvertebrate communities at the 13 study beaches from 2009 to 2011, conducting a total of 35 surveys, primarily in the late summer and fall (Table 1). Surveys were conducted during spring low tides in daylight when surface activity of invertebrates is minimal. We used sediment cores to collect samples, which were sieved through 1.5 mm mesh to retain macrofauna. Two different sampling designs were used, one similar to the sampling design in the 1970s survey period with a sample area that was proportional to and varied with active intertidal width and one with a fixed sampling area that was independent of active intertidal width (Table 1; Appendix S1; Fig. S1; Schooler, Dugan, & Hubbard, 2014). We chose to employ two survey methods in order to better calibrate for differences in sampling design and effort between survey periods (Schooler et al., 2014). The sampling design that was independent of active intertidal width and surveyed the most area is the preferred method by beach ecologists (Schlacher et al., 2008). Using this method to maximize sampling effort in the 2009–11 surveys allowed a more robust evaluation of change in species richness between survey periods.

### 2.2 Beach characteristics

Physical attributes and macrophyte wrack are considered to be the key drivers of community structure on beaches, far more important than biological interactions (Brown & McLachlan, 2010; Dugan et al., 2003; McLachlan, Jaramillo, Donn, & Wessels, 1993). For each survey, we measured active intertidal widths and collected sand samples for grain size analysis. Data on sand grain size and active intertidal beach width from fall surveys (August–November) for study beaches in the 1970s were extracted from Patterson, Straughan, and handwritten field and laboratory data sheets.

In the 1970s, sand samples were collected every 3.0 m along the basepoint transect spanning the width of the active intertidal. In 2009–11, sand samples were collected on each transect at the 24‐hr high tide line and water table outcrop, standard locations for calculating mean grain size (Brown & McLachlan, 2010). Sand was rinsed with fresh water, dried, and run through graded sieves in the laboratory. We calculated arithmetic mean grain size for each sand sample from the 2009‐11 surveys using the R package “G2Sd” (Gallon & Fournier, 2013). We measured the abundance of wrack as cover in the 2009–11 surveys using the methods of Dugan et al. (2003) which employs a line intercept method along each transect sampled for macroinvertebrates. A number of the physical characteristics recorded in the 1970s surveys were limited and often qualitative. Therefore, it was not possible to make quantitative temporal comparisons of wrack cover and morphodynamic state across the survey periods.

### 2.3 Data analysis

#### 2.3.1 Evaluating change in biodiversity

To estimate cumulative richness from the 2009‐11 surveys, we used rarefaction (when 1970s sample area < 2009–11) or extrapolation (1970s sample area > 2009–11) methods of Colwell et al. (2012) and Chao et al. (2014) to adjust observed species richness for differences in sampling areas among survey periods (hereafter adjusted species richness). Species–area curves with 95% confidence limits were generated for each site for the 2009–11 surveys using EstimateS software (version 9.0; Colwell, 2013), both for the overall intertidal community and wrack‐associated macroinvertebrates. Where multiple sampling designs were employed in the recent surveys (nine beaches), we used data from the sampling design that yielded the greatest area sampled across the 2009–11 surveys to create species–area curves. Generally this was the fixed area design (eight of 13 beaches).

To compare adjusted species richness between sampling periods, we plotted the cumulative species number versus cumulative area sampled from the 1970s surveys on the species–area curves constructed from 2009‐11 biodiversity data for each beach. The position of the value for the 1970s cumulative species richness and area with respect to the 95% confidence intervals of the species–area curves was used to assess the direction, magnitude, and significance of differences in species richness between time periods. We estimated the adjusted species richness from the 2009–11 surveys as the point on the species–area curve at the cumulative area sampled in the 1970s. The direction and magnitude of the difference in species richness between the survey periods was estimated by calculating the difference between the cumulative species richness from each 1970s survey and the estimated adjusted species richness from the 2009–11 surveys at the same beach for two categories of macroinvertebrates, overall and wrack‐associated taxa.

We used these results to evaluate patterns in the direction and magnitude of change in species richness between survey periods. Change in richness was expressed as a percentage with positive and negative values representing increases and decreases in species richness over time, respectively.

#### 2.3.2 Drivers of intertidal richness

Differences in active intertidal width and mean grain size between the 1970s and 2009–11 survey periods were evaluated with a one‐way ANOVA (SPSS v.17.0) and also expressed as percentages. We used OLS regression to evaluate relationships among mean grain size, active intertidal width, and wrack cover (2009–11 only) with the overall and wrack‐associated species richness adjusted for sampling area for our 2009–11 surveys (SPSS v.17.0). We inspected the residuals for these model regressions visually by using standard diagnostic plots to assess violations of model assumptions. Bearing in mind that sample sizes were small (*n *=* *13), we found little evidence of heteroscedasticity, trends, or non‐normality among residuals.

To evaluate impacts from climate change or other processes operating on regional spatial scales, we looked for widespread declines in species richness across study beaches consistent with large‐scale environmental drivers (e.g., sea level rise, sea surface temperature [SST], wave height). To assess influence of local scale processes on species richness over time, we compiled information on disturbance activities (Table 1) and beach characteristics for each study beach. We evaluated responses of species richness to these potential regional and local drivers for three categories of macroinvertebrates: overall, wrack‐associated, and low dispersal wrack‐associated species.

## 3 RESULTS

### 3.1 Biodiversity

**Figure 3:**
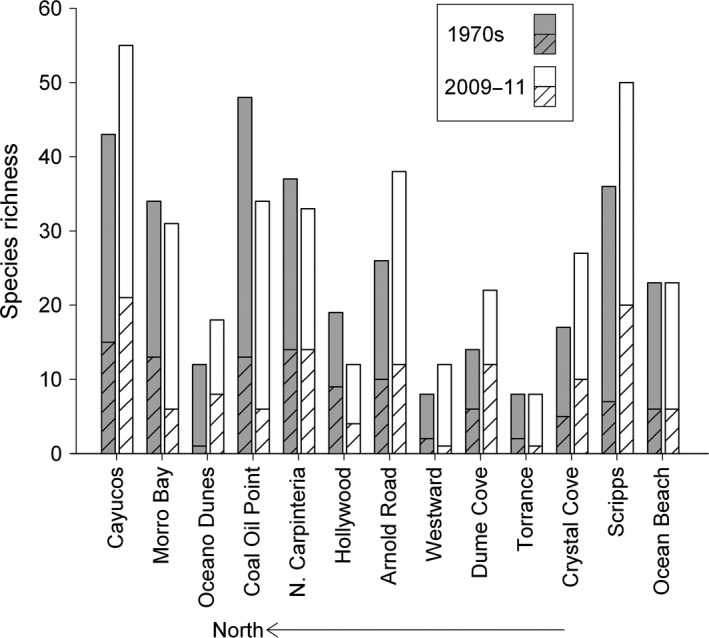
Values of cumulative observed species richness of macroinvertebrates at the study beaches for the 1970s and 2009–11 survey periods. The hatched bars indicate the values of observed richness for wrack‐associated species for each survey period. Species richness values are not adjusted for sampling

A total of 109 species (89 total species in each survey period) of intertidal macroinvertebrates (*x*– = 36.2 ± 5.0 *SE* species per site, *n *=* *13, range = 11–64 species) were recorded in all beach surveys (249 surveys). No regional patterns in observed species richness were evident in either survey period (Figure 3). Observed species richness varied > sixfold across the 13 study beaches but was similar in the two survey periods (1970s: 8–48 species per site, 2009–11: 8–55 species per site) (Figure 3).

In both survey periods, intertidal macroinvertebrate communities were dominated by Polychaeta (33%), Insecta (28%), and Crustacea (27%). Only one species, the sand crab *Emerita analoga*, was collected at every beach in both survey periods (Table S1). Other common taxa include the peracarid isopod, *Excirolana* sp., and the polychaetes, *Hemipodia* sp., *Nephtys californiensis*, and *Scolelepis bullibranchia*, which were collected at ≥ 11 of the 13 study beaches (Table S1). Two species, the talitrid amphipod, *Megalorchestia columbiana* and gastropod, *Callianax biplicata*, were collected at slightly less than half of the beaches in the 1970s and only one or two beaches in the 2009–11 surveys, the largest decline in occurrence we observed for species that were collected in both survey periods (Table S1).

Wrack‐associated invertebrates made up greater than a third of the total observed macroinvertebrate species in each survey period (1970s: 34%, 2009–11: 39%) (Figure 3). Species lacking planktonic larval stages and with limited dispersal abilities as adults made up more than a third of these wrack‐associated taxa (1970s: 37%; 2009–11: 35%) and 12% of the total species (1970s: 13%; 2009–11: 14%). Eight wrack‐associated species of peracarid Crustacea, all of which brood their young and have limited adult dispersal, were found including six species of talitrid amphipods (*Megalorchestia* spp.) and two oniscid isopods (*Alloniscus perconvexus* and *Tylos punctatus*) (Table 2; Table S1). At least one species of talitrid amphipod was collected at every beach with the exception of Westward (Table S1). Oniscid isopods were more restricted in distribution (nine beaches) (Table S1). Coleoptera were the most diverse order of wrack‐associated macroinvertebrates with 24 species from six families observed across the survey periods at our study beaches (Table S1). Of these, four species are flightless with low dispersal ability (Table 2). Overall species richness of Coleoptera increased across the survey periods with five more species observed in 2009–11 (Table S1).

**Table 2:** (a) Wrack‐associated species with low dispersal known from the study region and (b) the cumulative number of genera and species collected at each study beach in each survey period

a. Low dispersal wrack‐associated species		
Crustacea	Insecta	
* *Talitridae:	* *Carabidae:	
* Megalorchestia benedicti*	* Akephorus marinus*	
* Megalorchestia californiana*	* *Curculionidae:	
* Megalorchestia columbiana*	* Emphyastes fucicola*	
* Megalorchestia corniculata*	* *Melyridae:	
* Megalorchestia minor*	* Endeodes* sp. (NC)	
* Megalorchestia pugettensis*	* *Staphylinidae:	
* *Alloniscidae:	* Thinopinus pictus*	
* Alloniscus perconvexus*	* Hadrotes crassus* (NC)	
* *Tylidae:	* *Tenibrionidae:	
* Tylos punctatus*	* Coelus ciliates*	
Arachnida	* Coelus globosus* (NC)	
* *Bdellidae:		
* Neomolgus littoralis*		

b. Number of low dispersal wrack‐associated genera and species collected				
Location	Genera		Species	
	1970s	2009–11	1970s	2009–11
Cayucos	3	4	6	7
Morro Bay	2	1	5	2
Oceano Dunes	**0**	**3**	**0**	**5**
Coal Oil Point	2	1	**5**	**1**
North Carpinteria	3	2	**5**	**3**
Hollywood	2	2	4	3
Arnold Road	3	4	6	6
Westward	0	0	0	0
Dume Cove	**2**	**5**	**4**	**8**
Torrance	0	1	0	1
Crystal Cove	2	3	4	5
Scripps	3	3	3	4
Ocean Beach	**3**	**1**	**3**	**1**

Differences of >1 genus or species across study periods are indicated in bold. NC = not collected in this study.

### 3.2 Beach characteristics

**Figure 4:**
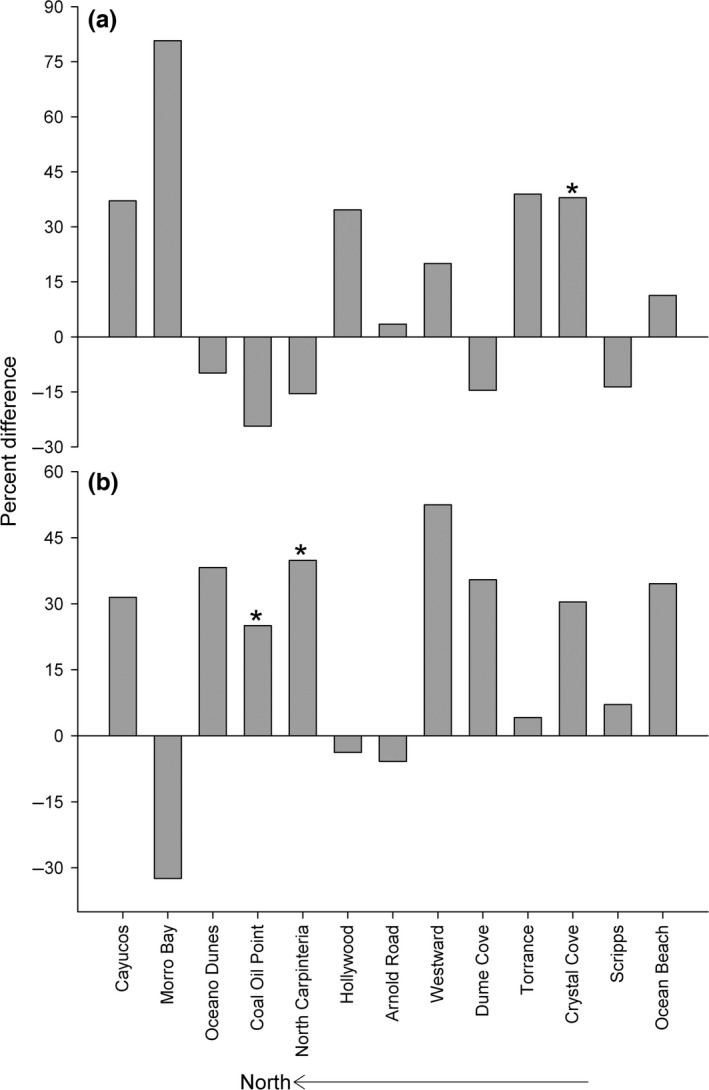
The direction of and percent difference observed in (a) mean active intertidal width and (b) mean grain size during fall (August–December) surveys for the study beaches between the 1970s and 2009–11 survey periods (* indicates sites where differences were significant at 0.05 level)

No regional patterns of change in beach characteristics were evident across survey periods. Active intertidal width and mean sand grain size remained similar between survey periods at the majority of study beaches and significant differences in these parameters were limited to three beaches (Figure 4, and Fig. S2). Many beaches in the study region, including four of our study beaches, are subject to intense anthropogenic impacts, such as grooming and beach fills, as part of the local coastal management regime (Table 1).

Mean values of active intertidal width, which represents habitat area available for beach communities, varied at least fivefold among study beaches in both survey periods, with values ranging from 24 m to 183 m across sites during fall surveys (Fig. S2a). A significant difference in mean active intertidal width between survey periods was detected at only one site, Crystal Cove (*F *=* *14.52, *p *=* *0.03), which was wider (38%) in the 2009–11 surveys (Figure 4a). The greatest loss of intertidal width between survey periods (24%) was observed at Coal Oil Point, but was not statistically significant (Figure 4a).

Mean values of sand grain size varied more than threefold among study beaches in both survey periods, ranging from 0.14 mm to 0.53 mm in fall surveys (Fig. S2b). Values for mean grain size were greater for the 2009–11 surveys than the 1970s surveys for the majority of beaches (10 beaches) and lower mean values were observed in the recent surveys at 25% of the beaches (three beaches) (Figure 4b). Significant differences in values of mean sand grain size between survey periods were detected at only two sites, Coal Oil Point (*F *=* *6.27, *p *= 0.03) and North Carpinteria (*F *=* *14.13, *p *= 0.03), with greater values in recent surveys for both beaches (Figure 4b).

The mean cover of macrophtye wrack varied more than 60‐fold (*x*– = 1.8 ± 0.5 m^2^ m^‐1^, range = 0.1–6.6 m^2^ m^‐1^) among study beaches in the 2009–11 surveys (Fig. S3). Ungroomed beaches (*x*– = 2.1 ± 0.7 m^2^ m^‐1^, *n *=* *9, range = 0.2–6.6 m^2^ m^‐1^) had four times the cover of wrack compared to urban beaches (*x*– = 0.5 ± 0.3 m^2^ m^‐1^, *n *=* *3, range = 0.1–1.1 m^2^ m^‐1^) with the exception of Ocean Beach which had high cover of fresh wrack (3.6 m^2^/m) in the lower intertidal zone (Fig. S3; Table 1).

### 3.3 Drivers of intertidal richness

#### 3.3.1 Local scale anthropogenic drivers

**Figure 5:**
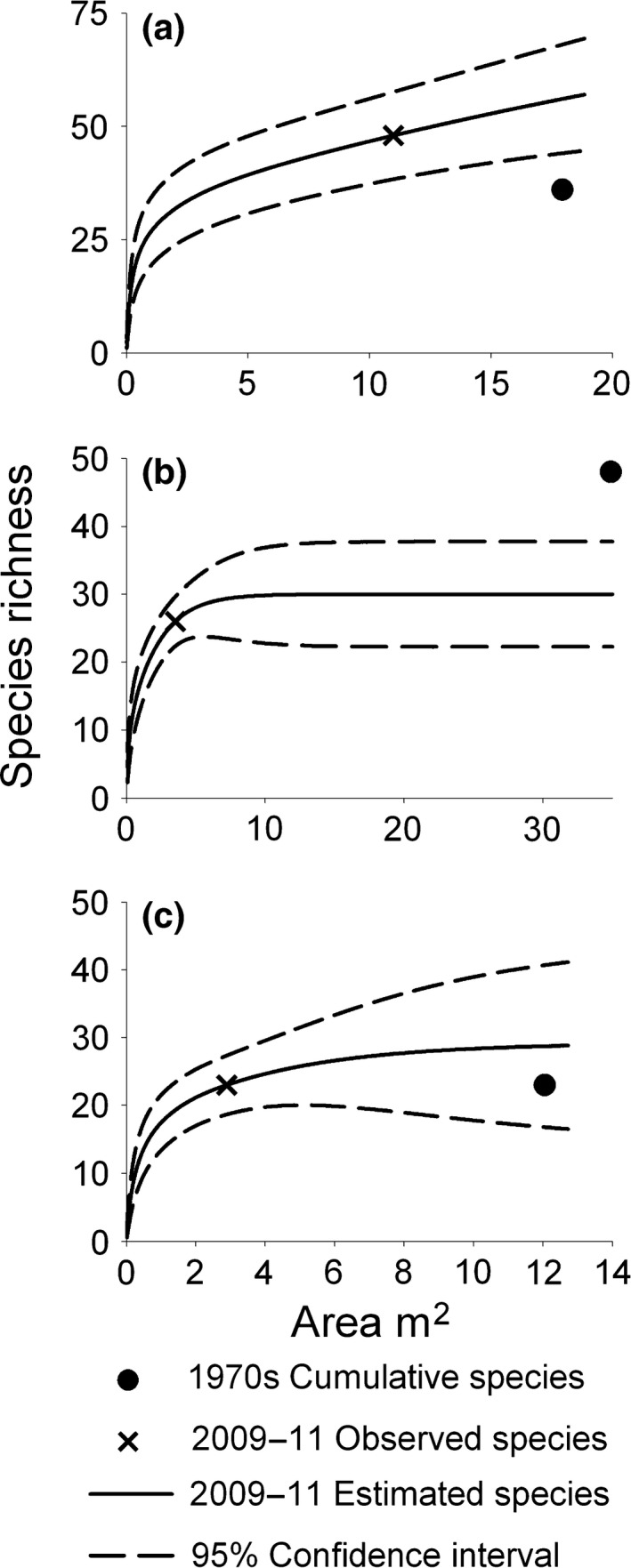
Example plots of species–area curves based on the 2009–11 surveys with unconditional 95% CI. The values of cumulative species number and sampling area in the 1970s surveys and the 95% CI of the 2009–11 species–area curves shown here were used to evaluate differences in species richness between survey periods. Adjusted species richness values were (a) significantly higher (Scripps), (b) significantly lower (Coal Oil Point), and (c) did not differ significantly (Ocean Beach) in 2009–11 compared to 1970s surveys. See Figs S4 and S5 in Supporting information for species–area curves for all study beaches

**Figure 6:**
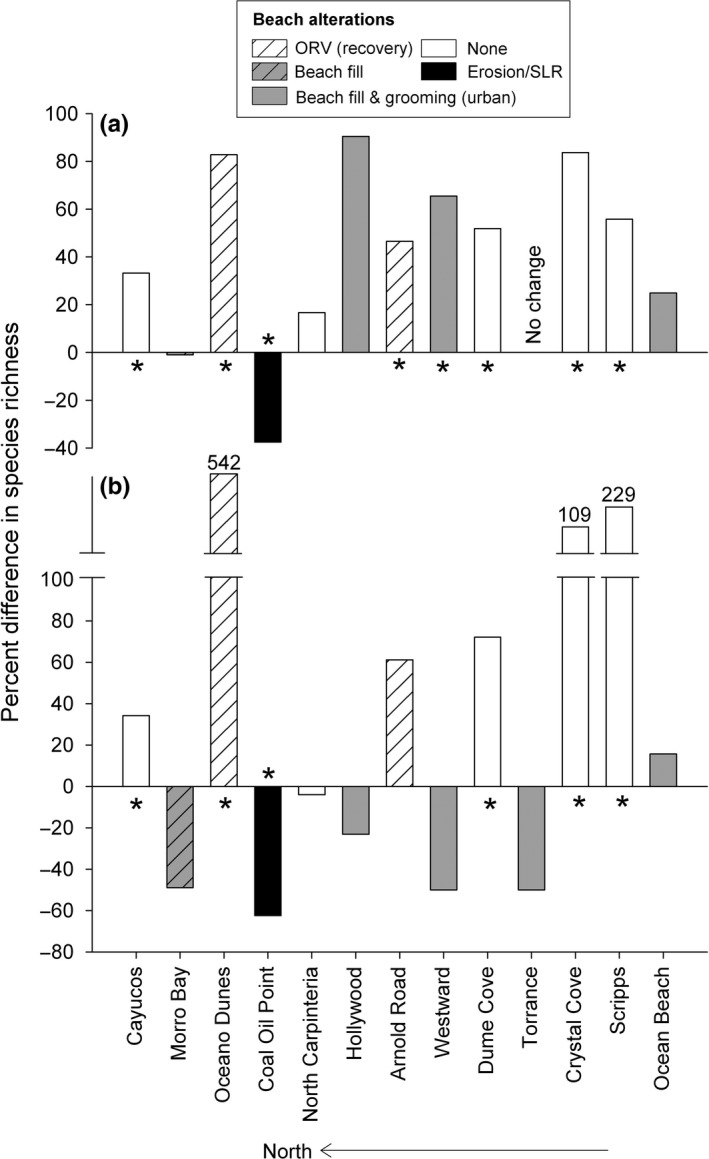
The direction of and percent difference reported in species richness between the 1970s and 2009–11 surveys for (a) adjusted overall species richness and (b) adjusted wrack‐associated species richness. White bars = no direct beach alteration detected. White hatched bars = change consistent with recovery from off‐road vehicle use. Gray bars = urban sites with continuous beach fills and grooming. Gray hatched bars = beach fill and upper‐beach habitat modification. Black bars = no direct beach alteration but overall and wrack‐associated adjusted richness declines were consistent with habitat loss from erosion and sea level rise (SLR). Asterisk = sites with significant (95% CI) differences in adjusted richness. See also Figs S4 and S5 in Supporting information

Although the multidecadal changes we detected in biodiversity varied in magnitude and direction among beaches, our analyses of values of adjusted species richness using species–area curves for overall and wrack‐associated species revealed several trends across the survey periods (Figures 5 and 6; see Figs S4 and S5 in Supporting information for details) that were related to the history of human impacts (Table 1). A large component of the differences we detected in adjusted species richness across the survey periods was driven by wrack‐associated species with ecologically important shifts in richness of this group evident for 77% (10) of the 13 beaches (Figure 6). Differences detected in the richness of wrack‐associated species exceeded those for overall adjusted species richness in magnitude at nine (69%) of the beaches and differed in direction at four (31%) of the beaches (Figure 6). For beaches with no detectable anthropogenic impacts, overall and wrack‐associated species richness increased significantly between the survey periods except for one beach where declines in species richness were consistent with habitat loss (Figure 6b; white and black bars with asterisks). Where local scale anthropogenic drivers were identified on our study beaches, we observed ecologically important declines in the species richness of the wrack‐associated and low dispersal wrack‐associated groups (Figure 6b; gray and gray hatched bars). For two beaches where ORV use was banned between survey periods, overall and wrack‐associated species richness increased in a manner that was consistent with recovery from impacts (Figure 6b; white hatched bars).

We also detected significant differences (Figure 5a,b) in overall and wrack‐associated adjusted richness between survey periods at five of six beaches where no direct beach alterations were identified during or between survey periods (Table 1) (Figure 6; white and black bars with asterisks). For these six beaches, cumulative overall and wrack‐associated species richness in the 1970s varied ≥ threefold among beaches (Figure 3). Higher values for overall and wrack‐associated adjusted richness were estimated in 2009–11 compared to 1970s surveys at four beaches (Figures 5a and 6; white bars). Adjusted overall richness ranged from 17% (Dume Cove) to 84% higher (Crystal Cove) with a mean value of 48% higher species richness (Figures 5a and 6a; white bars). In contrast, for one beach (Coal Oil Point), overall and wrack‐associated adjusted richness was much lower (38% and 62%, respectively) in the 2009–11 surveys compared to the 1970s (Figures 5b and 6; black bars), a result consistent with the observed loss of intertidal habitat estimated by intertidal width (Figure 4). Notably, four species of talitrid amphipods with low dispersal abilities were reported at Coal Oil Point in the 1970s surveys compared to only one species in the 2009–11 surveys, a 75% decline in richness of this ecologically important genus (Table 2).

For degraded urban beaches that experienced continuous impacts from intensive grooming and major beach fills (Table 1) which commenced well before the 1970s surveys and continued throughout both survey periods, the adjusted richness of wrack‐associated species declined despite increases in overall richness at three of four beaches (Figure 6b; gray bars). Values of cumulative overall and wrack‐associated species richness in the 1970s were low and varied threefold and fourfold, respectively, among these beaches (Figure 3). Overall adjusted richness at these four beaches was 45% higher on average in the 2009–11 surveys (Figure 6a; gray bars). However, at three of these beaches, the average value for adjusted richness of wrack‐associated species was 41% lower in the 2009–11 surveys (Hollywood: 23%, Westward: 50%, and Torrance: 50%; Figure 6b; gray bars). For wrack‐associated species with low dispersal abilities, major differences in the number of species between survey periods were also evident at the fourth urban beach, Ocean Beach (Table 2). Two taxa, a talitrid amphipod and an oniscid isopod*,* recorded in the 1970s surveys were not detected at Ocean Beach in the 2009 or 2010 surveys, representing a loss of two genera, the greatest loss observed for any study beach despite gains in overall and wrack‐associated adjusted richness (Table 2).

Beach fills conducted during and between the survey periods at one beach, Morro Bay, included large dredge pipes stretched across upper‐beach habitat and our sampling area during the 2009 survey (Table 1). Despite these direct impacts, cumulative overall richness was high (34 species; Figure 4) in the 1970s and little change (<1%) in overall adjusted richness was estimated between the survey periods (Figures 5c and 6a; gray hatched bars). In contrast, we observed declines (49%) for wrack‐associated adjusted richness at this beach similar to that observed at two degraded urban beaches (50% at Westward and Torrance) (Figure 6b; gray hatched bars). Moreover, a 60% decline in observed richness of wrack‐associated species with low dispersal ability was evident at this site (Table 2).

Our results on intertidal richness were consistent with recovery from intense ORV use at two beaches, Oceano Dunes and Arnold Road, where overall and wrack‐associated adjusted richness were higher in the 2009–11 surveys (Figures 5a and 6; white hatched bars). Both beaches were subject to intense ORV traffic during the 1970s (Table 1), but management changes prohibited ORVs about 15 years prior to our 2009–11 surveys (1984 at Oceano Dunes and 1992 at Arnold Road). Overall adjusted richness was significantly higher in the 2009–11 surveys at both beaches, but was only statistically significant for wrack‐associated species at Oceano Dunes (Figures 5a and 6; white hatched bars). Wrack‐associated species with low dispersal not previously recorded in the 1970s surveys were detected in our 2009–11 surveys at both beaches, including talitrid amphipods at Oceano Dunes and oniscid isopods at Arnold Road and Oceano Dunes (Table 2). At Oceano Dunes, three genera and five species of wrack‐associated invertebrates not observed in the 1970s were found in our 2009–11 surveys (Table 2), the largest increase we observed in these taxa with low dispersal.

#### 3.3.2 Environmental drivers

The responses of species richness to beach characteristics, including active intertidal width, sand grain size, and wrack abundance, were not consistent across groups and survey periods. Overall adjusted species richness was negatively related to sand grain size in both survey periods (1970s: *r* ^2^ = 0.38, *p *= 0.02, *n *=* *13; 2009–11: *r* ^2^ = 0.47, *p *= 0.01, *n *=* *13), as was the adjusted richness of wrack‐associated biota for the 2009–11 surveys (*r* ^2^ = 0.30, *p *= 0.05, *n *=* *13) (Fig. S6). Relationships between mean active intertidal width and (1) overall species richness and (2) the richness of wrack‐associated species were positive but not significant for both periods. Across the survey periods, we found no relationships between the observed change in overall or wrack‐associated adjusted species richness and those in beach width or grain size for the 13 study beaches. For 2009–11 surveys, the adjusted overall (*r* ^2^ = 0.31, *p *= 0.05, *n *=* *13) and wrack‐associated (*r* ^2^ = 0.27, *p *= 0.07, *n *=* *13) species richness were positively related to the mean cover of wrack indicating the importance of this resource to biodiversity (Fig. S7).

## 4 DISCUSSION

Wildlife populations that rely on sandy beaches as critical habitat, such as sea turtles and nesting plovers, are threatened worldwide, largely in response to human disturbance and habitat alteration (e.g., Schlacher et al., 2014). Our results documenting the loss of sandy beach invertebrate species that depend on macrophyte wrack, particularly those with limited dispersal, strongly echo trends observed for beach‐dependent wildlife on urban coasts and highlight the importance on local scale processes. Disturbed beaches continued to lose species over more than three decades, while beaches recovering from impacts slowly gained species.

We observed an overall lack of regional declines in species richness and change in physical beach characteristics despite regional increases in SST, sea level rise, wave height, and storm frequency across the survey periods (Allan & Komar, 2006; NOAA Tides & Currents, 2017; Ruggiero, Komar, & Allan, 2010; Smith et al., 2006). This suggests that processes operating at local scales are exerting a stronger influence on sandy beach biodiversity than regional or global scale drivers and highlight the importance of human impacts at local spatial scales. Our results are broadly consistent with studies across a range of ecosystems and communities showing that local and regional scale changes observed in biodiversity often do not clearly correspond with global biodiversity loss (Dornelas et al., 2014; Elahi et al., 2015; Hautekèete et al., 2015; Sax & Gaines, 2003; Thomas, 2013; Vellend et al., 2013). At a global scale, change in species number is the difference between the speciation and extinction rates, and increasing extinction rates have resulted in a net global loss of species over the last century (Sax & Gaines, 2003). At smaller spatial scales, immigration and emigration drive metacommunity dynamics that can obscure the signal of species extinctions at broader scales (Gonzalez et al., 2016; Sax & Gaines, 2003). Our results demonstrate why a long‐term and ecologically informed perspective is needed to reveal declines in biodiversity that may not be apparent in more broad‐brush analyses (Gonzalez et al., 2016).

The influence of local and regional processes on sandy beach biodiversity contrasts with results for rocky shores for the same eastern Pacific region over a similar time span (Smith et al., 2006). In rocky intertidal communities associated with mussels, fewer intertidal species of invertebrates were observed in 2002 compared to the 1960s and 1970s at sites across California (Smith et al., 2006). Based on these results, Smith et al. (2006) concluded that the consistency of trends for observed species richness in intertidal mussel beds among sites suggested that large‐scale processes impacted species richness over the past 30 years despite spatially varying intensities of human use and pollution across the study region. They attributed these changes to climate change‐driven warming and decreased phytoplankton productivity lowering food supply to mussel beds and decreasing pelagic larval survival. Although our study covered similar regions and time spans, we did not observe analogous local or regional declines in intertidal species richness for sandy beach communities. One potential factor in the disparate results for these two intertidal ecosystems is the relative importance of the wrack‐associated fauna to intertidal biodiversity on beaches, compared to suspension‐feeding animals that depend on phytoplankton. This diverse functional group lacks planktonic larval stages and largely depends on beached giant kelp (*Macrocystis pyrifera*) and other marine macrophytes from nearby kelp forests for food and shelter (Dugan et al., 2003; Lastra, Page, Dugan, Hubbard, & Rodil, 2008). Although giant kelp biomass has shown considerable local scale variability over the past three decades, no consistent regional patterns have emerged (Bell, Cavanaugh, Reed, & Siegel, 2015). Our results showing that change in wrack‐associated species richness occurred over time but the direction and magnitude of that change varied strongly among beaches are consistent with local scale resource availability and human impacts as drivers of diversity change rather than regional changes in productivity or pelagic larval survival as proposed for rocky shores.

Increases in species richness can be associated with higher connectivity caused by declines in regional barriers and the immigration or introduction of exotic species (Sax & Gaines, 2003). However, the higher richness of macroinvertebrate species we found in the 2009–11 surveys at approximately half of the study beaches was not due to the presence of exotic species. Although a small number of cryptogenic sandy beach macroinvertebrate species were found in our surveys (<5% on average) during both survey periods, no known non‐native intertidal species were observed. Globally, few exotic species of macroinvertebrates have been reported for sandy beach ecosystems (Defeo et al., 2009). This result contrasts with temperate regions and coastal ecosystems in general (Cohen & Carlton, 1998; Heard, Sax, & Bruno, 2012), which are often considered hot spots for biological invasions (Ruiz, Fofonoff, Steves, Foss, & Shiba, 2011). Our finding of no exotic or invasive intertidal invertebrates at beaches representing a wide range in biodiversity and intensity of disturbance for a widespread coastal ecosystem suggests that the invasion potential may be smaller for beaches than other ecosystems.

Our results of significant increases in overall species richness over more than 30 years at four relatively unmanaged beaches as well as three impacted beaches were unexpected and indicate further study of these dynamic communities and the local drivers affecting biodiversity, including coastal management, are warranted. For two of the beaches, the positive response in diversity was consistent with recovery from disturbance by ORV use. Similar increases in species richness found at the five other study beaches may have also responded to changes in coastal management across the survey periods, including the past establishment of marine reserves at Scripps and Crystal Cove. However, it is not possible to quantitatively evaluate these unexpected changes using our datasets and the limited information available on past coastal management activities. Monitoring of the biodiversity of sandy beach ecosystems that takes advantage of the recent establishment of a widespread (~1,350 km) network of marine protected areas overlapping the study region could provide an opportunity to gain insights into factors underlying the long‐term changes in biodiversity we identified here.

Long‐term changes we detected in richness differed substantially among functional groups of intertidal invertebrates. In particular, comparisons of overall species richness alone failed to capture the significant loss of a key functional group, the wrack‐associated species, in sandy beach ecosystems across three decades. Wrack‐associated taxa play a vital role in the breakdown and processing of macroalgal wrack and subsequent nutrient cycling on beaches (Dugan, Hubbard, Page, & Schimel, 2011; Lastra et al., 2008) and are important prey for wildlife, particularly shorebirds including endangered species (Dugan et al., 2003). The lack of planktonic larval stages and the low dispersal abilities of some adult invertebrates (Table 2a) make wrack‐associated species especially sensitive to anthropogenic impacts that directly affect their populations or the abundance of wrack subsidies that provide food and shelter (Hubbard et al., 2014). Removal or disturbance of wrack resources has been shown to strongly impact wrack‐associated taxa and diversity (Dugan et al., 2003; Llewellyn & Shackley, 1996). The changes in richness of wrack‐associated species we observed were consistent with the impacts of wrack removal and associated disturbance at four urban beaches subject to regular beach grooming (Hollywood, Westward, Torrance, and Ocean Beach) and one beach with heavy upper‐beach habitat modification (beach fill and dredge pipes) during our surveys (Morro Bay). Despite a result of higher or no change in overall adjusted species richness values at these beaches, wrack‐associated richness had declined by greater than 20% at all but one groomed beach in 2009–11 compared to the 1970s. Similarly, ORVs can severely impact wrack as well as dune vegetation and biota (Davies, Speldewinde, & Stewart, 2016; Schlacher & Thompson, 2008). At two of our study beaches that were subject to intense ORV traffic during the 1970s, biodiversity increased across the survey periods likely in response to the elimination of ORV disturbance more than 15 years before our 2009–11 surveys. In particular, richness of wrack‐associated species, including five species with low dispersal never recorded in the 1970s, increased, indicating that recovery of these sensitive species may be possible given adequate time and sufficient local source populations.

Beach habitat loss associated with erosion and sea level rise is expected to result in declines in intertidal biodiversity where retreat of beaches is constrained by coastal bluffs or armoring (Dugan et al., 2008, 2013; Jaramillo et al., 2012; Schoeman et al., 2014). Our results were consistent with this prediction at only one site, Coal Oil Point, a narrow beach backed by a coastal bluff. There, both overall and wrack‐associated species richness declined (38% and 62%, respectively) by 2009–11, concomitant with a 24% decrease in the active intertidal width, a 25% increase in mean sand grain size, and a 1.11 mm/year increase in local monthly mean sea level (data from Santa Barbara Station 1973–2015; NOAA Tides & Currents, 2017). This loss and alteration of intertidal beach habitat likely resulted from a combination of sea level rise and climate forcing that increased wave height and storm frequency (Allan & Komar, 2006; Ruggiero et al., 2010), such as the Pacific Decadal Oscillation and El Niño‐Southern Oscillation (Barnard, Hubbard, & Dugan, 2012; Mantua, Hare, Zhang, Wallace, & Francis, 1997; Revell et al., 2011) and anthropogenic impacts to sediment supply (Barnard et al., 2012; Orme et al., 2011; Revell & Griggs, 2006). These factors, combined with the very limited scope for retreat due to the coastal bluff bounding this beach (Dugan et al., 2013), may explain why observed declines in species richness were so striking here compared to the other study beaches. Narrow beaches constrained by bluffs or armoring are common in central and southern California but are at risk of complete habitat loss from erosion and sea level rise (Vitousek et al., 2017). By 2100, 31%–67% of all southern California beaches are projected to disappear due to erosion (Vitousek et al., 2017). These results illustrate regional (e.g., sea level rise and wave height) and local (e.g., sand starvation and armoring) processes that threaten biodiversity and ecosystem integrity of this widespread coastal ecosystem in the region and around the globe (Dugan et al., 2008, 2010; Schlacher et al., 2007; Schoeman et al., 2014).

The need for robust evaluations of shifts in biodiversity driven by climate and anthropogenic drivers at relevant temporal and spatial scales to inform conservation and management is growing (Magurran et al., 2010; Sternberg & Yakir, 2015). We were able to overcome differences in sampling effort and methodologies associated with comparisons of biodiversity across multidecadal temporal scales using an extrapolation approach (Chao et al., 2014; Colwell et al., 2012; Schooler et al., 2014). Using cumulative species richness values obtained from species lists to compare area‐adjusted species richness across survey periods made it possible for us to identify and evaluate ecologically relevant differences in intertidal biodiversity over a multidecadal time scale. Greater use of such methods could allow much needed temporal comparisons of biodiversity to be made in ecosystems with datasets that might otherwise be unsuitable for comparison, particularly underrepresented biomes and ecosystems outside of developed nations, to accurately quantify global biodiversity change (Gonzalez et al., 2016).

Our results highlight how the complexity of ecologically important change in biodiversity can be masked in comparisons of overall species richness (Elahi et al., 2015). Identifying taxa and functional groups known to be vulnerable to specific impacts can be an important tool to inform *a priori* predictions of biodiversity change. Despite detecting declines in overall species richness at only one study beach, we found that ecologically important wrack‐associated fauna vulnerable to disturbance, resource limitation, and habitat loss declined at numerous beaches, suggesting considerable alteration of the structure and function of a major coastal ecosystem has occurred in the region over three decades. We also found evidence that local impacts to this ecologically important component of intertidal biodiversity may be reversed with management changes that reduce disturbance of beaches and allow recovery of sensitive taxa. Shifts in species composition and loss or gain of key taxa, such as those we observed for wrack‐associated species, can be as important as overall biodiversity change when the taxa are critical to ecosystem function (Hooper et al., 2012; Lefcheck et al., 2015; Orwin, Ostle, Wilby, & Bardgett, 2014). Our findings illustrate the value of using detailed long‐term comparisons of community composition at local and regional scales in evaluating the status of biodiversity. These types of evaluations can inform policies intended to conserve and manage the endemic biodiversity of beaches and other vulnerable ecosystems**.**

## CONFLICT OF INTEREST

None declared.

## AUTHOR CONTRIBUTIONS

NKS, JED, and DMH designed the study. DS conducted 1970s surveys and provided data. NKS, JED, and DMH conducted 2009–2011 surveys. NKS processed samples and analyzed data with JED. NKS, JED, and DMH wrote the manuscript.

## References

[ece33064-bib-0001] Allan, J. C. , & Komar, P. D. (2006). Climate controls on US West Coast erosion processes. Journal of Coastal Research, 22, 511–529.

[ece33064-bib-0002] Barnard, P. L. , Hubbard, D. M. , & Dugan, J. E. (2012). Beach response dynamics of a littoral cell using a 17‐year single‐point time series of sand thickness. Geomorphology, 139, 588–598.

[ece33064-bib-0003] Bell, T. W. , Cavanaugh, K. C. , Reed, D. C. , & Siegel, D. A. (2015). Geographical variability in the controls of giant kelp biomass dynamics. Journal of Biogeography, 42, 2010–2021.

[ece33064-bib-0004] Brown, A. C. , & McLachlan, A. (2010). The ecology of sandy shores. Burlington, MA: Academic Press.

[ece33064-bib-0005] Bruno, J. F. , Kennedy, C. W. , Rand, T. A. , & Grant, M. B. (2004). Landscape‐scale patterns of biological invasions in shoreline plant communities. Oikos, 107, 531–540.

[ece33064-bib-0006] Burrows, M. T. , Schoeman, D. S. , Buckley, L. B. , Moore, P. , Poloczanska, E. S. , Brander, K. M. , … Richardson, A. J. (2011). The pace of shifting climate in marine and terrestrial ecosystems. Science, 334, 652–655. 22053045 10.1126/science.1210288

[ece33064-bib-0007] Butchart, S. H. , Walpole, M. , Collen, B. , van Strien, A. , Scharlemann, J. P. , Almond, R. E. , … Watson, R. (2010). Global biodiversity: Indicators of recent declines. Science, 328, 1164–1168. 20430971 10.1126/science.1187512

[ece33064-bib-0008] Cardinale, B. J. , Duffy, J. E. , Gonzalez, A. , Hooper, D. U. , Perrings, C. , Venail, P. , … Naeem, S. (2012). Biodiversity loss and its impact on humanity. Nature, 486, 59–67. 22678280 10.1038/nature11148

[ece33064-bib-0009] Chao, A. , Gotelli, N. , Hsieh, T. C. , Sander, E. , Ma, K. H. , Colwell, R. K. , & Ellison, A. M. (2014). Rarefaction and extrapolation with Hill numbers: A framework for sampling and estimation in species diversity studies. Ecological Monographs, 84, 45–67.

[ece33064-bib-0010] Chen, I. C. , Hill, J. K. , Ohlemüller, R. , Roy, D. B. , & Thomas, C. D. (2011). Rapid range shifts of species associated with high levels of climate warming. Science, 333, 1024–1026. 21852500 10.1126/science.1206432

[ece33064-bib-0011] Cohen, A. N. , & Carlton, J. T. (1998). Accelerating invasion rate in a highly invaded estuary. Science, 279, 555–558. 9438847 10.1126/science.279.5350.555

[ece33064-bib-0012] Colwell, R. K. (2013). EstimateS: Statistical estimation of species richness and shared species from samples. Version 9.0. User's Guide and application published at: http://purl.oclc.org/estimates

[ece33064-bib-0013] Colwell, R. K. , Chao, A. , Gotelli, N. J. , Lin, S. Y. , Mao, C. X. , Chazdon, R. L. , & Longino, J. T. (2012). Models and estimators linking individual‐based and sample‐based rarefaction, extrapolation and comparison of assemblages. Journal of Plant Ecology, 5, 3–21.

[ece33064-bib-0014] Davies, R. , Speldewinde, P. C. , & Stewart, B. A. (2016). Low level off‐road vehicle (ORV) traffic negatively impacts macroinvertebrate assemblages at sandy beaches in south‐western Australia. Scientific Reports, 6, 24899. 27121212 10.1038/srep24899 PMC4848469

[ece33064-bib-0015] Defeo, O. , McLachlan, A. , Schoeman, D. S. , Schlacher, T. A. , Dugan, J. , Jones, A. , … Scapini, F. (2009). Threats to sandy beach ecosystems: A review. Estuarine, Coastal and Shelf Science, 81, 1–12.

[ece33064-bib-0016] Devictor, V. , & Robert, A. (2009). Measuring community responses to large‐scale disturbance in conservation biogeography. Diversity and Distributions, 15, 122–130.

[ece33064-bib-0017] Dornelas, M. , Gotelli, N. J. , McGill, B. , Shimadzu, H. , Moyes, F. , Sievers, C. , & Magurran, A. E. (2014). Assemblage time series reveal biodiversity change but not systematic loss. Science, 344, 296–299. 24744374 10.1126/science.1248484

[ece33064-bib-0018] Dugan, J. E. , Defeo, O. , Jaramillo, E. , Jones, A. R. , Lastra, M. , Nel, R. , … Schoeman, D. S. (2010). Give beach ecosystems their day in the sun. Science, 329, 1146. 10.1126/science.329.5996.1146-a 20813935

[ece33064-bib-0020] Dugan, J. E. , Hubbard, D. M. , McCrary, M. D. , & Pierson, M. O. (2003). The response of macrofauna communities and shorebirds to macrophyte wrack subsidies on exposed sandy beaches of southern California. Estuarine, Coastal and Shelf Science, 58, 25–40.

[ece33064-bib-0021] Dugan, J. E. , Hubbard, D. M. , Page, H. M. , & Schimel, J. P. (2011). Marine macrophyte wrack inputs and dissolved nutrients in beach sands. Estuaries and Coasts, 34, 839–850.

[ece33064-bib-0022] Dugan, J. E. , Hubbard, D. M. , & Quigley, B. J. (2013). Beyond beach width: Steps toward identifying and integrating ecological envelopes with geomorphic features and datums for sandy beach ecosystems. Geomorphology, 199, 95–105.

[ece33064-bib-0023] Dugan, J. E. , Hubbard, D. M. , Rodil, I. F. , Revell, D. L. , & Schroeter, S. (2008). Ecological effects of coastal armoring on sandy beaches. Marine Ecology, 29, 160–170.

[ece33064-bib-0024] Elahi, R. , O'Connor, M. I. , Byrnes, J. E. , Dunic, J. , Eriksson, B. K. , Hensel, M. J. , & Kearns, P. J. (2015). Recent trends in local‐scale marine biodiversity reflect community structure and human impacts. Current Biology, 25, 1938–1943. 26166784 10.1016/j.cub.2015.05.030

[ece33064-bib-0025] Gallon, R. K. , & Fournier, J. (2013). G2Sd: Grain‐size statistics and description of sediment, R package version 2.0. Retrieved from http://cran.r-project.org/web/packages/G2Sd/index.html,

[ece33064-bib-0026] Gamfeldt, L. , Lefcheck, J. S. , Byrnes, J. E. , Cardinale, B. J. , Duffy, J. E. , & Griffin, J. N. (2015). Marine biodiversity and ecosystem functioning: What's known and what's next? Oikos, 124, 252–265.

[ece33064-bib-0027] Gonzalez, A. , Cardinale, B. J. , Allington, G. R. , Byrnes, J. , Endsley, K. A. , Brown, D. G. , … Loreau, M. (2016). Estimating local biodiversity change: A critique of papers claiming no net loss of local diversity. Ecology, 97, 1949–1960. 27859190 10.1890/15-1759.1

[ece33064-bib-0028] Grantham, B. A. , Eckert, G. L. , & Shanks, A. L. (2003). Dispersal potential of marine invertebrates in diverse habitats. Ecological Applications, 13, 108–116.

[ece33064-bib-0029] Harley, C. D. G. , Randall Hughes, A. , Hultgren, K. M. , Miner, B. G. , Sorte, C. J. B. , Thornber, C. S. , … Williams, S. L. (2006). The impacts of climate change in coastal marine systems. Ecology Letters, 9, 228–241. 16958887 10.1111/j.1461-0248.2005.00871.x

[ece33064-bib-0030] Hautekèete, N. C. , Frachon, L. , Luczak, C. , Toussaint, B. , Van Landuyt, W. , Van Rossum, F. , & Piquot, Y. (2015). Habitat type shapes long‐term plant biodiversity budgets in two densely populated regions in north‐western Europe. Diversity and Distributions, 21, 631–642.

[ece33064-bib-0031] Heard, M. J. , Sax, D. F. , & Bruno, J. F. (2012). Dominance of non‐native species increases over time in a historically invaded strandline community. Diversity and Distributions, 18, 1232–1242.

[ece33064-bib-0032] Hoegh‐Guldberg, O. , & Bruno, J. F. (2010). The impact of climate change on the world's marine ecosystems. Science, 328, 1523–1528. 20558709 10.1126/science.1189930

[ece33064-bib-0033] Hooper, D. U. , Adair, E. C. , Cardinale, B. J. , Byrnes, J. E. , Hungate, B. A. , Matulich, J. L. , … O'Connor, M. I. (2012). A global synthesis reveals biodiversity loss as a major driver of ecosystem change. Nature, 486, 105–108. 22678289 10.1038/nature11118

[ece33064-bib-0034] Hubbard, D. M. , Dugan, J. E. , Schooler, N. K. , & Viola, S. M. (2014). Local extirpations and regional declines of endemic upper beach invertebrates in southern California. Estuarine, Coastal and Shelf Science, 150, 67–75.

[ece33064-bib-0035] Jaramillo, E. , Dugan, J. E. , Hubbard, D. M. , Melnick, D. , Manzano, M. , Duarte, C. , … Sanchez, R. (2012). Ecological implications of extreme events: Footprints of the 2010 earthquake along the Chilean coast. PLoS One, 7, e35348. 22567101 10.1371/journal.pone.0035348 PMC3342270

[ece33064-bib-0036] Lastra, M. , Page, H. M. , Dugan, J. E. , Hubbard, D. M. , & Rodil, I. F. (2008). Processing of allochthonous macrophyte subsidies by sandy beach consumers: Estimates of feeding rates and impacts on food resources. Marine Biology, 154, 163–174.

[ece33064-bib-0037] Lefcheck, J. S. , Byrnes, J. E. , Isbell, F. , Gamfeldt, L. , Griffin, J. N. , Eisenhauer, N. , … Duffy, J. E. (2015). Biodiversity enhances ecosystem multifunctionality across trophic levels and habitats. Nature Communications, 6, 1–7. 10.1038/ncomms7936 PMC4423209 25907115

[ece33064-bib-0038] Llewellyn, P. J. , & Shackley, S. E. (1996). The effects of mechanical beach‐cleaning on invertebrate populations. British Wildlife, 7, 147–155.

[ece33064-bib-0039] Magurran, A. E. , Baillie, S. R. , Buckland, S. T. , Dick, J. M. , Elston, D. A. , Scott, E. M. , … Watt, A. D. (2010). Long‐term datasets in biodiversity research and monitoring: Assessing change in ecological communities through time. Trends in Ecology & Evolution, 25, 574–582. 20656371 10.1016/j.tree.2010.06.016

[ece33064-bib-0040] Mantua, N. J. , Hare, S. R. , Zhang, Y. , Wallace, J. M. , & Francis, R. C. (1997). A Pacific interdecadal climate oscillation with impacts on salmon production. Bulletin of the American Meteorological Society, 78, 1069–1079.

[ece33064-bib-0041] McLachlan, A. , Jaramillo, E. , Defeo, O. , Dugan, J. , de Ruyck, A. , & Coetzee, P. (1995). Adaptations of bivalves to different beach types. Journal of Experimental Marine Biology and Ecology, 187, 147–160.

[ece33064-bib-0042] McLachlan, A. , Jaramillo, E. , Donn, T. E. , & Wessels, F. (1993). Sandy beach macrofauna communities and their control by the physical environment: A geographical comparison. Journal of Coastal Research, 15, 27–38.

[ece33064-bib-0043] NOAA Tides & Currents . (2017). NOAA sea level trends. Retrieved from https://tidesandcurrents.noaa.gov/sltrends/sltrends.html

[ece33064-bib-0044] Novoa, A. , Talley, T. S. , Talley, D. M. , Crooks, J. A. , & Reyns, N. B. (2016). Spatial and temporal examination of bivalve communities in several estuaries of Southern California and Northern Baja California, MX. PLoS One, 11, e0148220. 26840744 10.1371/journal.pone.0148220 PMC4740503

[ece33064-bib-0045] Orme, A. R. , Griggs, G. B. , Revell, D. L. , Zoulas, J. G. , Grandy, C. C. , & Koo, H. (2011). Beach changes along the southern California coast in the 20th century: A comparison of natural and human forcing factors. Shore & Beach, 79, 38–50.

[ece33064-bib-0046] Orwin, K. H. , Ostle, N. , Wilby, A. , & Bardgett, R. D. (2014). Effects of species evenness and dominant species identity on multiple ecosystem functions in model grassland communities. Oecologia, 174, 979–992. 24213721 10.1007/s00442-013-2814-5

[ece33064-bib-0047] Pacifici, M. , Foden, W. B. , Visconti, P. , Watson, J. E. , Butchart, S. H. , Kovacs, K. M. , … Rondinini, C. (2015). Assessing species vulnerability to climate change. Nature Climate Change, 5, 215–224.

[ece33064-bib-0048] Parmesan, C. , & Yohe, G. (2003). A globally coherent fingerprint of climate change impacts across natural systems. Nature, 421, 37–42. 12511946 10.1038/nature01286

[ece33064-bib-0049] Patterson, M. M. (1974). Intertidal macrobiology of selected sandy beaches in southern California. Report, Allan Hancock Foundation and Institute for Marine and Coastal Studies, University of Southern California, Los Angeles.

[ece33064-bib-0050] Pimm, S. L. , Russell, G. J. , Gittleman, J. L. , & Brooks, T. M. (1995). The future of biodiversity. Science, 269, 347–349. 17841251 10.1126/science.269.5222.347

[ece33064-bib-0051] Revell, D. L. , Dugan, J. E. , & Hubbard, D. M. (2011). Physical and ecological responses of sandy beaches to the 1997–98 El Niño. Journal of Coast Research, 27, 718–730.

[ece33064-bib-0052] Revell, D. L. , & Griggs, G. B. (2006). Beach width and climate oscillations along Isla Vista, Santa Barbara, California. Shore & Beach, 74, 8–16.

[ece33064-bib-0053] Ruggiero, P. , Komar, P. D. , & Allan, J. C. (2010). Increasing wave heights and extreme value projections: The wave climate of the US Pacific Northwest. Coastal Engineering, 57, 539–552.

[ece33064-bib-0054] Ruiz, G. M. , Fofonoff, P. W. , Steves, B. , Foss, S. F. , & Shiba, S. N. (2011). Marine invasion history and vector analysis of California: A hotspot for western North America. Diversity and Distributions, 17, 362–373.

[ece33064-bib-0055] Sala, O. E. , Chapin, F. S. , Armesto, J. J. , Berlow, E. , Bloomfield, J. , Dirzo, R. , … Wall, D. H. (2000). Global biodiversity scenarios for the year 2100. Science, 287, 1770–1774. 10710299 10.1126/science.287.5459.1770

[ece33064-bib-0056] Sax, D. F. , & Gaines, S. D. (2003). Species diversity: From global decreases to local increases. Trends in Ecology & Evolution, 18, 561–566.

[ece33064-bib-0057] Schiel, D. R. , & Foster, M. S. (2015). The biology and ecology of giant kelp forests. Oakland, CA: University of California Press.

[ece33064-bib-0058] Schlacher, T. A. , Dugan, J. , Schoeman, D. S. , Lastra, M. , Jones, A. , Scapini, F. , … Defeo, O. (2007). Sandy beaches at the brink. Diversity and Distributions, 13, 556–560.

[ece33064-bib-0059] Schlacher, T. A. , Jones, A. R. , Dugan, J. E. , Weston, M. A. , Harris, L. R. , Schoeman, D. S. , … Peterson, C. H. (2014). Open‐coast sandy beaches and coastal dunes In Lockwood J., & Masalo B. (Eds.), Coastal conservation (pp. 37–94). Cambridge: Cambridge University Press.

[ece33064-bib-0060] Schlacher, T. A. , Schoeman, D. S. , Dugan, J. , Lastra, M. , Jones, A. , Scapini, F. , & McLachlan, A. (2008). Sandy beach ecosystems: Key features, sampling issues, management challenges and climate change impacts. Marine Ecology, 29, 70–90.

[ece33064-bib-0061] Schlacher, T. A. , & Thompson, L. M. C. (2008). Physical impacts caused by off‐road vehicles (ORVs) to sandy beaches: Spatial quantification of car tracks on an Australian barrier island. Journal of Coastal Restoration, 24, 234–242.

[ece33064-bib-0062] Schoeman, D. S. , Schlacher, T. A. , & Defeo, O. (2014). Climate‐change impacts on sandy‐beach biota: Crossing a line in the sand. Global Change Biology, 20, 2383–2392. 25121188 10.1111/gcb.12505

[ece33064-bib-0063] Schooler, N. K. , Dugan, J. E. , & Hubbard, D. M. (2014). Detecting change in intertidal species richness on sandy beaches: Calibrating across sampling designs. Estuarine, Coastal and Shelf Science, 150, 58–66.

[ece33064-bib-0064] Smith, J. R. , Fong, P. , & Ambrose, R. F. (2006). Dramatic declines in mussel bed community diversity: Response to climate change? Ecology, 87, 1153–1161. 16761594 10.1890/0012-9658(2006)87[1153:ddimbc]2.0.co;2

[ece33064-bib-0065] Sternberg, M. , & Yakir, D. (2015). Coordinated approaches for studying long‐term ecosystem responses to global change. Oecologia, 177, 921–924. 25676103 10.1007/s00442-015-3237-2

[ece33064-bib-0066] Straughan, D. (1982). Inventory of the natural resources of sandy beaches in Southern California. Report, Allan Hancock Foundation and Institute for Marine and Coastal Studies, University of Southern California, Los Angeles.

[ece33064-bib-0067] Thomas, C. D. (2013). Local diversity stays about the same, regional diversity increases, and global diversity declines. Proceedings of the National Academy of Sciences of the United States of America, 110, 19187–19188. 24248347 10.1073/pnas.1319304110 PMC3845126

[ece33064-bib-0068] Tilman, D. , Isbell, F. , & Cowles, J. M. (2014). Biodiversity and ecosystem functioning. Annual Review of Ecology, Evolution, and Systematics, 45, 471–493.

[ece33064-bib-0069] Vellend, M. , Baeten, L. , Myers‐Smith, I. H. , Elmendorf, S. C. , Beauséjour, R. , Brown, C. D. , … Wipf, S. M. (2013). Global meta‐analysis reveals no net change in local‐scale plant biodiversity over time. Proceedings of the National Academy of Sciences of the United States of America, 110, 19456–19459. 24167259 10.1073/pnas.1312779110 PMC3845118

[ece33064-bib-0070] Vitousek, S. , Barnard, P. L. , Limber, P. , Erikson, L. , & Cole, B. (2017). A model integrating long shore and cross‐shore for predicting long‐term shoreline responses to climate change. Journal of Geophysical Research: Earth Surface, 122, 782–806.

[ece33064-bib-0071] Zabin, C. J. , Danner, E. M. , Baumgartner, E. P. , Spafford, D. , Miller, K. A. , & Pearse, J. S. (2013). A comparison of intertidal species richness and composition between Central California and Oahu, Hawaii. Marine Ecology, 34, 131–156.

