# Supplementary material for: Local scale processes drive long‐term change in biodiversity of sandy beach ecosystems
Source: Ecol Evol. 2017 May 25;7(13):4822–34. doi: 10.1002/ece3.3064 (PMC5496535; doi:10.1002/ece3.3064)
Supplement: Supplementary file 2 [file ECE3-7-4822-s002.docx]

**Supporting Information**

Local scale processes drive long-term change in biodiversity of sandy beach ecosystems

Nicholas K. Schooler, Jenifer E. Dugan, David M. Hubbard, and Dale Straughan

*Ecology & Evolution*

**Appendix S1.** Detailed methods for the 1970s and 2009-11 biodiversity surveys.

1970s biodiversity surveys:

Basepoints described by Straughan (1982) for each site were used to locate the start of a transect line that was laid out from the basepoint to the water’s edge (low swash) at low tide (Fig. S1). Surveys were conducted during spring low tides year round. Study beaches were only selected if they were surveyed during the late summer or fall. Samples were collected using cores or quadrats along this transect spanning the active intertidal zone (24 h high tide line to the low swash) employing a variety of sampling designs (Straughan, 1982). Samples were sieved through 1.5 mm mesh to retain macroinvertebrates for counting and identification.

2009-11 biodiversity surveys:

At each site, we stretched a transect tape from the same basepoint used by Straughan (1982) (Table 1) in the 1970s to the low swash (Fig. S1). The proportional area surveys consisted of a contiguous grid of 3.0 x 3.0 m quadrats in four shore-normal columns and y along-shore rows (y = active intertidal width/3.0 m) that spanned the active intertidal. Within each quadrat, two sediment cores (10 cm diameter, 20 cm depth) were randomly collected and pooled as a sample. The fixed area (3.5 m^2^) survey design was similar to that used in intertidal surveys conducted by Dugan *et al*. (2003). We collected 150 cores at uniform intervals along each of three shore-normal transects randomly spaced along-shore (450 cores per site) from the 24 h high tide line to the low swash. Cores were pooled in contiguous groups of 10 to make 15 total samples per transect. Slightly less area was sampled at Dume Cove and Crystal Cove (Table 1) using the fixed area survey design because samples collected landward of the 24 h high tide line were excluded. Each pooled core sample was sieved through 1.5 mm mesh to remove sand and retain macrofauna, placed in labeled plastic bags, and returned to the laboratory where they were preserved in 10% buffered formalin. Macroinvertebrates were counted and identified to the lowest taxonomic level possible, typically species. Inconsistencies in taxonomic identification and nomenclature between study periods were addressed by consulting the literature and taxonomic specialists. Species that do not normally inhabit beaches or are considered parasitic were excluded from our analyses.

References:

Dugan, J. E., Hubbard, D. M., McCrary, M. D. & Pierson, M. O. (2003) The response of macrofauna communities and shorebirds to macrophyte wrack subsidies on exposed sandy beaches of southern California. *Estuarine, Coastal and Shelf Science*, **58**, 25–40.

Straughan, D. (1982) *Inventory of the natural resources of sandy beaches in Southern California*. Report, Allan Hancock Foundation and Institute for Marine and Coastal Studies, University of Southern California, Los Angeles.


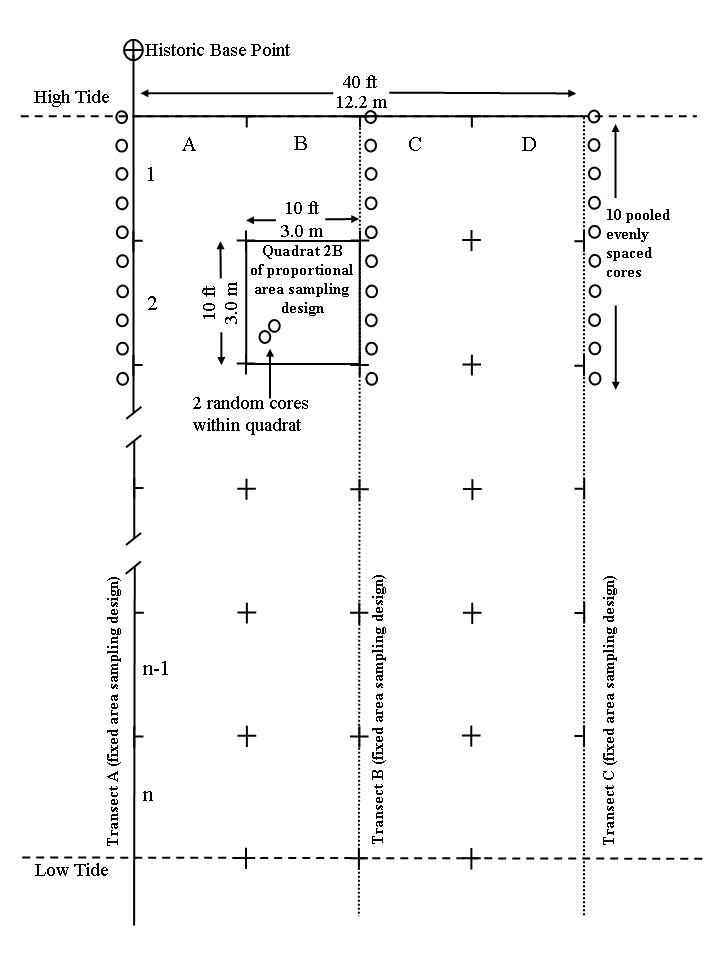


**Figure S1**. Diagram of the layout for the proportional area sampling design and the fixed area sampling design relative to the historic baseline transect, 24 h high tide line (high tide), and low swash (low tide). Figure is not drawn to scale. The diagram was first published by Schooler *et al*. (2014) and permission for reuse was given by Elsevier.

Schooler, N. K., Dugan, J. E. & Hubbard, D. M. (2014) Detecting change in intertidal species richness on sandy beaches: calibrating across sampling designs. *Estuarine, Coastal and Shelf Science*, **150**, 58–66.


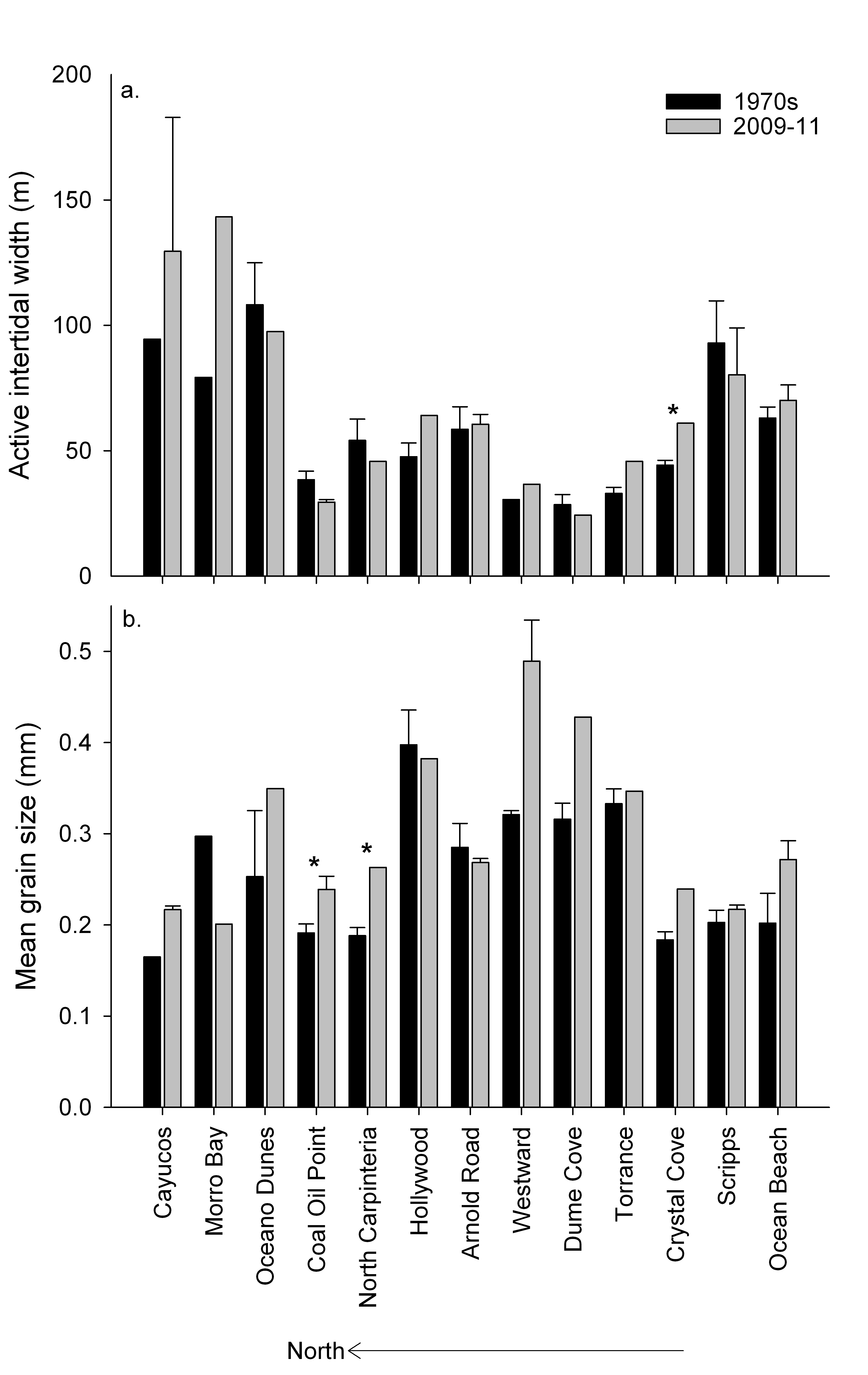


**Figure** **S2.** Mean values (± 1 SE) for a) active intertidal width and b) mean grain size during fall (August-December) surveys for beaches in the 1970s and 2009-11 (* indicates significant differences (*P* ≤ 0.05)). SEs are not reported for sites where only one survey was conducted in 2009-11 except for Westward where active intertidal width was the same in both the 2009 and 2010 surveys.


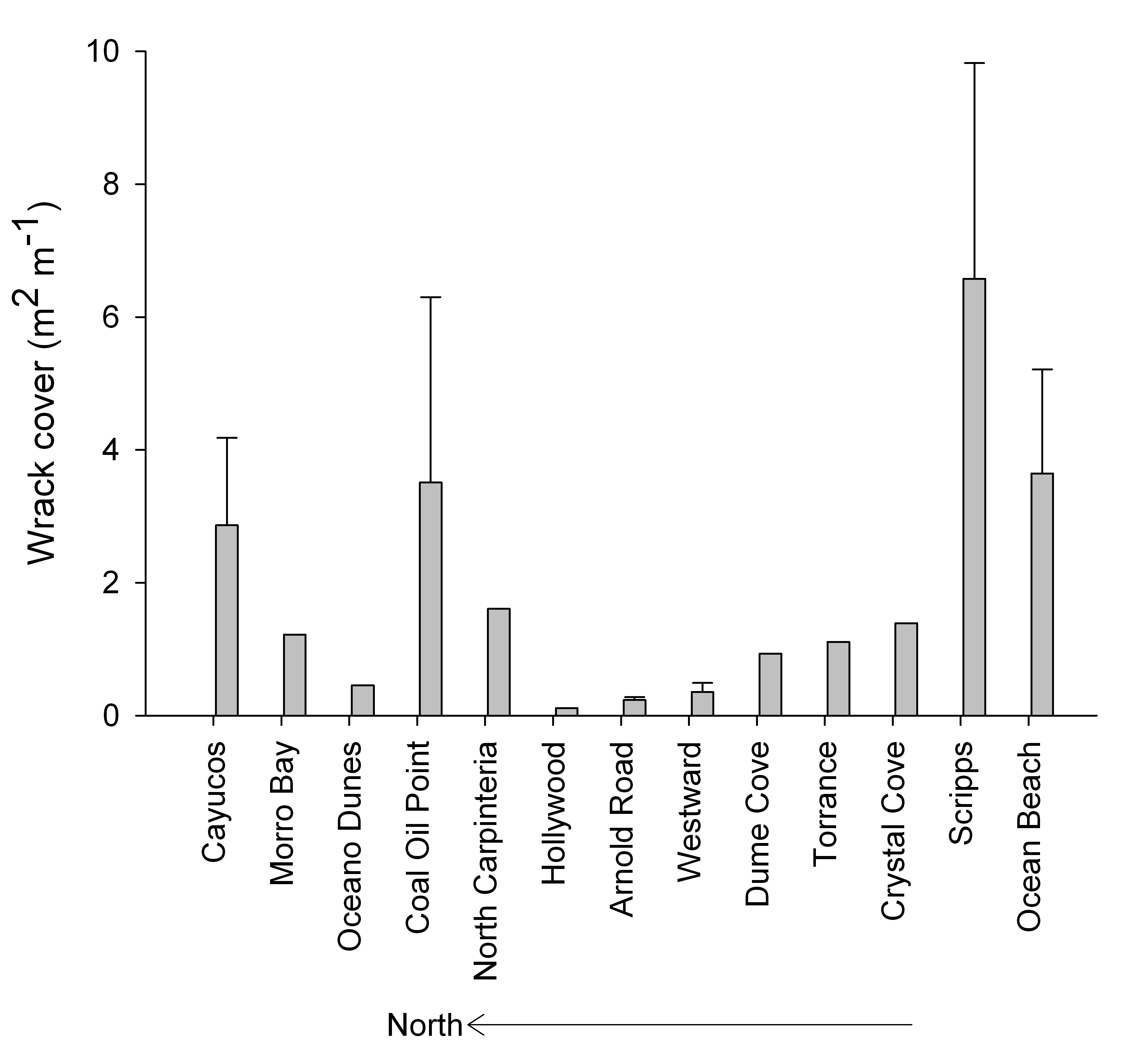


**Figure** **S3.** Mean values (± 1 SE) for wrack cover for beaches in the 2009-11 surveys. SEs are not reported for sites where only one survey was conducted in 2009-11.


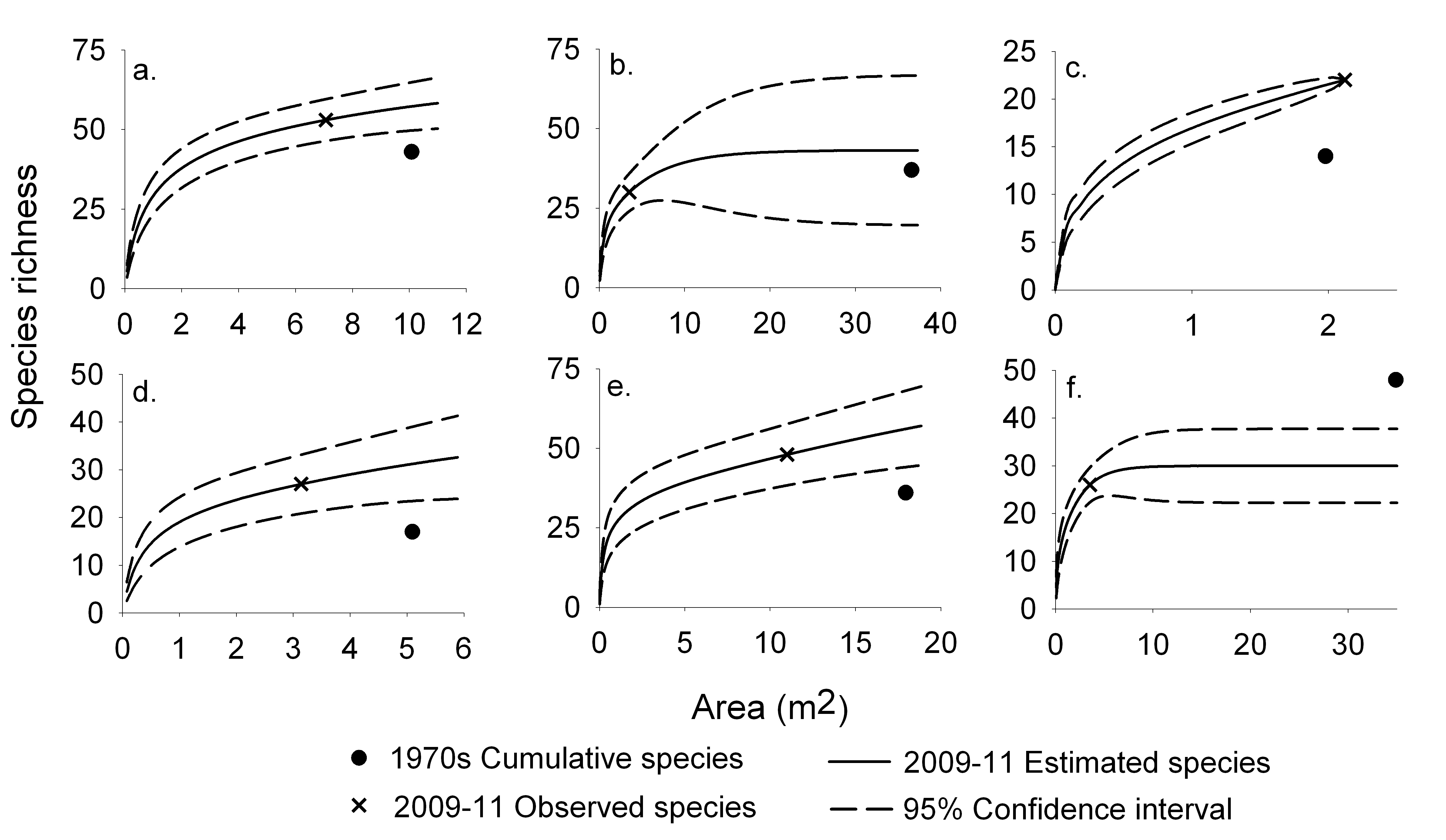


**Figure S4.** Plots of species-area curves based on the 2009-2011 surveys with unconditional 95% CI for six beaches where no direct beach alteration during or between survey periods were detected. The values of cumulative species number and sampling area in the 1970s surveys and the 95% CI of the 2009-2011 species area curves shown here were used to evaluate differences in species richness between survey periods. The plots are organized by increases or decreases in richness between periods and geography. Species richness values were higher in the 2009-11 surveys at a) Cayucos, b) North Carpinteria, c) Dume Cove, d) Crystal Cove, and e) Scripps and lower in the 2009-11 surveys at f) Coal Oil Point.


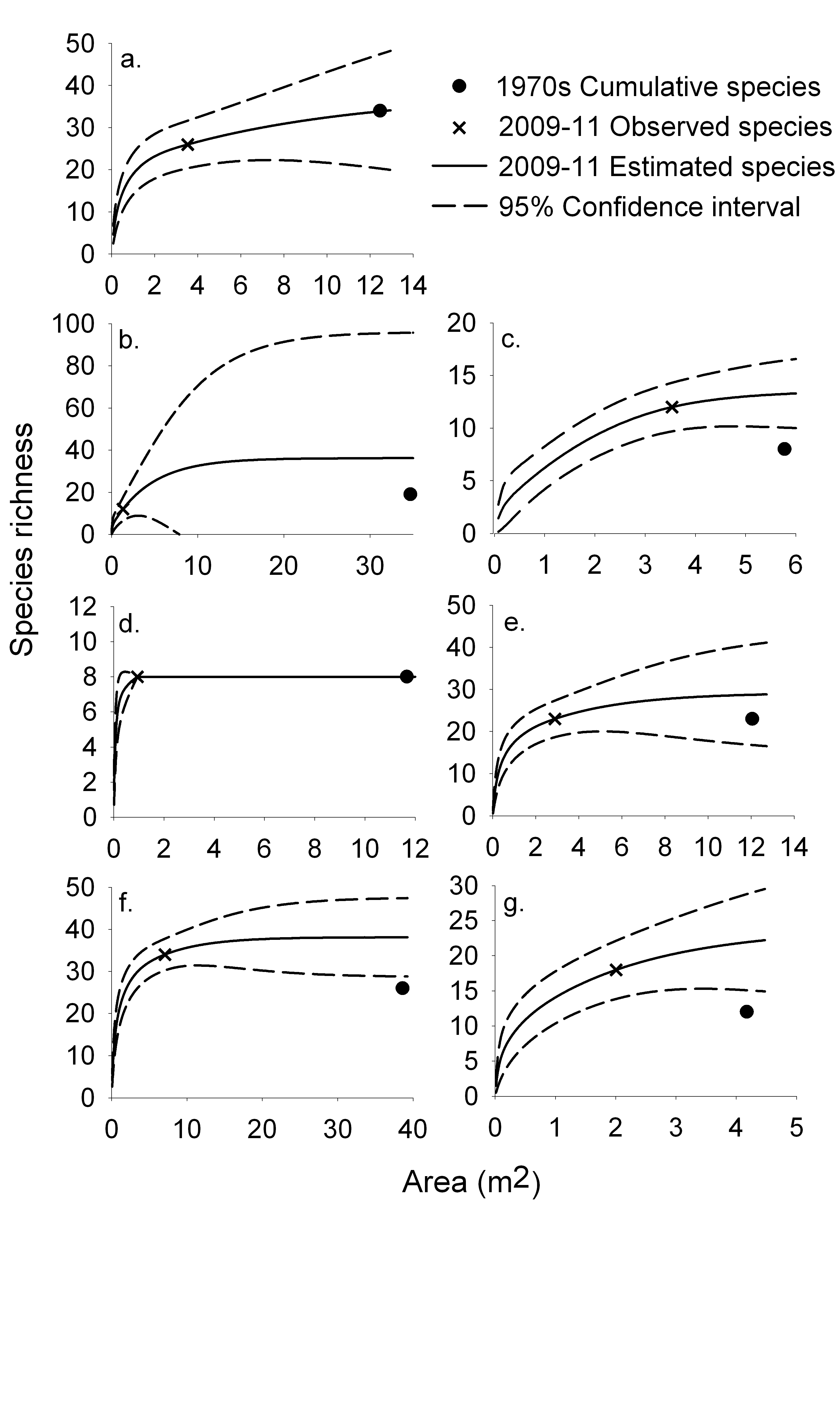


**Figure S5.** Plots of species-area curves based on the 2009-2011 surveys with unconditional 95% CI for seven beaches where direct beach alteration during or between survey periods were detected. The cumulative species number and sampling area in the 1970s surveys and the 95% CI of the 2009-2011 species area curves shown here were used to evaluate differences in species richness between the survey periods. The plots are organized by direct alteration type. Beach fill and upper beach alteration occurred at a) Morro Bay. Beach fills and grooming occurred at b) Hollywood, c) Westward, d) Torrance, and e) Ocean Beach. Off-road vehicle use occurred during the 1970s surveys at f) Arnold Rd and g) Oceano Dunes.

^
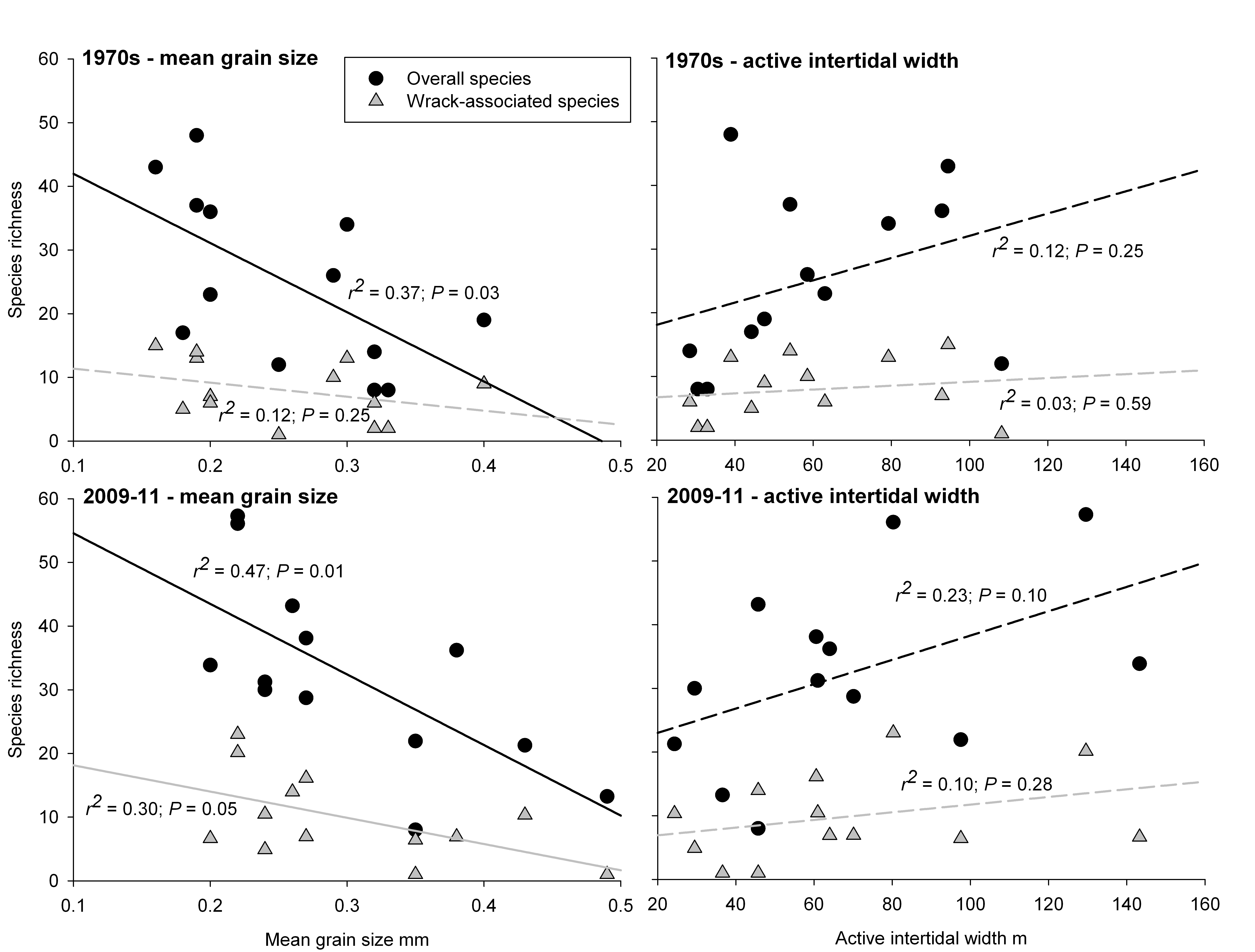
^

**Figure S6.** The relationships between overall (black) and wrack-associated (gray) species richness as a function of mean grain size (left) and active intertidal width (right) for the 1970s (top) and 2009-11 (bottom) surveys. The regression correlation coefficients and *P*-values are displayed on the graphs adjacent to the lines with which they correspond. Relationships that are significant (*P* ≤ 0.05) are indicated by a solid line.

^
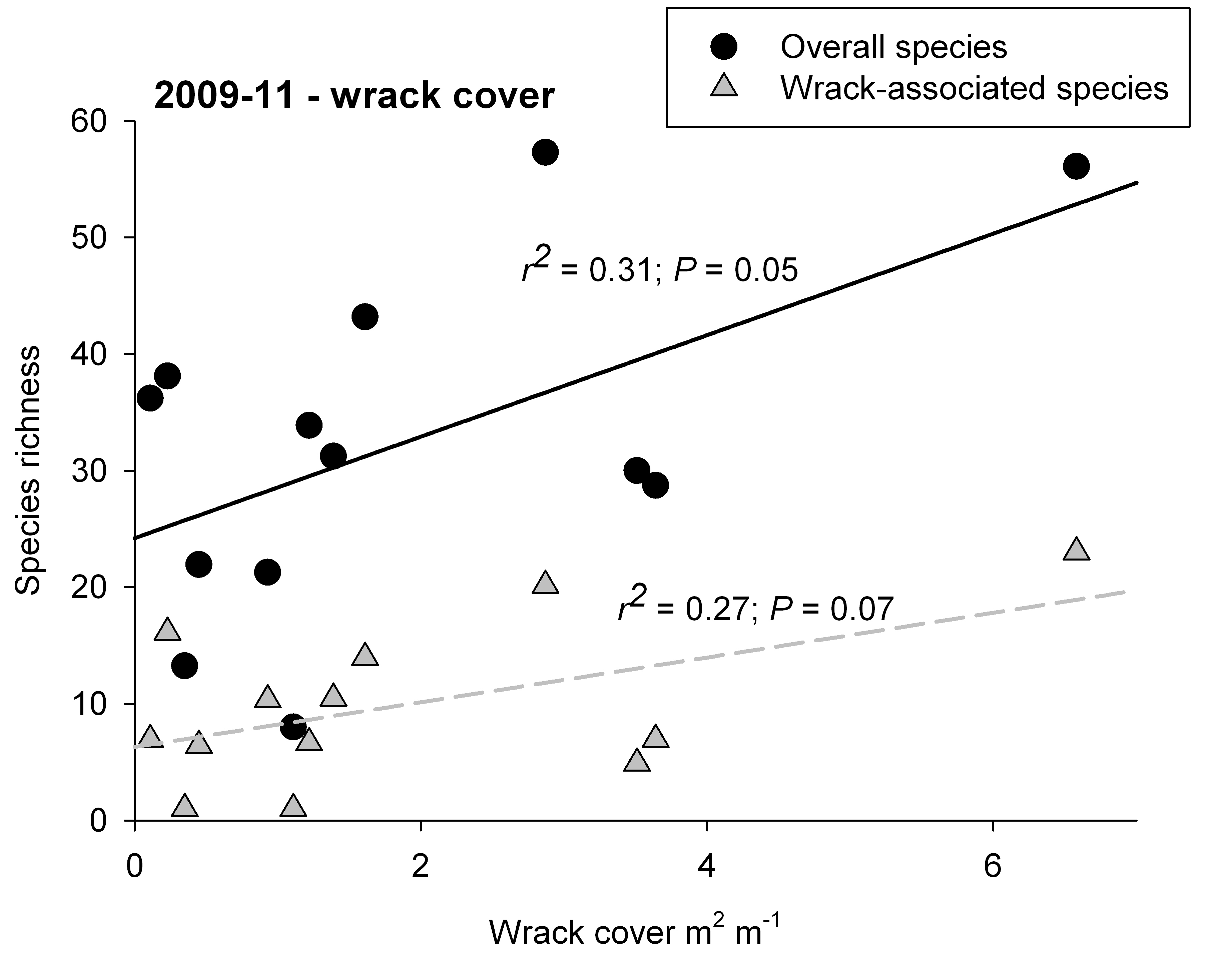
^

**Figure S7.** The relationships between overall (black) and wrack-associated (gray) species richness as a function of wrack cover. The regression correlation coefficients and *P*-values are displayed on the graphs adjacent to the lines with which they correspond. Relationships that are significant (*P* ≤ 0.05) are indicated by a solid line.
